# Optimizing a digital intervention for managing blood pressure in stroke patients using a diverse sample: Integrating the person‐based approach and patient and public involvement

**DOI:** 10.1111/hex.13173

**Published:** 2020-12-14

**Authors:** Tanvi Rai, Kate Morton, Cristian Roman, Roisin Doogue, Cathy Rice, Marney Williams, Claire Schwartz, Carmelo Velardo, Lionel Tarassenko, Lucy Yardley, Richard J. McManus, Lisa Hinton

**Affiliations:** ^1^ Nuffield Department of Primary Care Health Sciences University of Oxford Oxford UK; ^2^ School of Psychology University of Southampton Southampton UK; ^3^ Department of Engineering Science University of Oxford UK; ^4^ Graduate Entry Medical School University of Limerick Limerick Ireland; ^5^ Public and Patient Involvement (PPI) Contributor Bristol UK; ^6^ Department of Experimental Psychology Institute of Biomedical Engineering University of Bristol Bristol UK; ^7^ The Healthcare Improvement Studies Institute University of Cambridge Cambridge UK

**Keywords:** blood pressure, community‐based recruitment, digital health, hypertension, patient and public involvement, person‐based approach, qualitative research, seldom‐heard groups, self‐monitoring, stroke

## Abstract

**Background:**

Having a stroke or transient ischaemic attack increases the risk of a subsequent one, especially with high blood pressure (BP). Home‐based BP management can be effective at maintaining optimal BP.

**Objective:**

To describe the optimization of a digital intervention for stroke patients and the value of participant diversity, using the person‐based approach (PBA) and integral patient and public involvement (PPI).

**Setting and participants:**

Stroke patients recruited from primary care and community settings, and health‐care professionals in primary care, in England and Ireland.

**Design:**

Three linked qualitative studies conducted iteratively to develop an intervention using the PBA, with integral PPI.

**Intervention:**

The BP: Together intervention, adapted from existing BP self‐monitoring interventions, is delivered via mobile phone or web interface to support self‐monitoring of BP at home. It alerts patients and their clinicians when a change in antihypertensive medication is needed.

**Findings:**

Feedback from a diverse range of participants identified potential barriers, which were addressed to improve the intervention accessibility, feasibility and persuasiveness. Easy‐to‐read materials were developed to improve usability for patients with aphasia and lower literacy. The importance of including family members who support patient care was also highlighted. Feedback messages regarding medication change were refined to ensure usefulness for patients and clinicians.

**Discussion:**

Input from PPI alongside qualitative research with a diverse study sample allowed the creation of a simple and equitable BP management intervention for stroke patients.

**Patient involvement:**

Two PPI co‐investigators contributed to design, conduct of study, data interpretation and manuscript preparation; community PPI sessions informed early planning. Study participants were stroke patients and family members.

## 1 INTRODUCTION

Stroke is the 4th leading cause of death in the UK, with more than 100 000 strokes or transient ischaemic attacks (TIA) occurring every year.^1^ Almost two‐thirds of people who have had a stroke leave hospital with a disability and/or suffer from limb impairment. Approximately one third are affected by aphasia, a complex language and communication disorder resulting from damage to the language centres of the brain.^1^ People from Black and minority ethnic groups (BAME), as well as those living in socially deprived areas, are at increased risk of hypertension‐related stroke.^2^, ^3^, ^4^ Having a stroke increases the risk of having a subsequent one, especially for those with high blood pressure (BP). Evidence suggests that effective BP control may be even more important for the prevention of secondary stroke than primary stroke^5^ and current guidelines recommend^6^ that antihypertensive treatment be ‘increased as quickly as tolerated’ for people who have had a stroke or TIA (hereafter referred to as ‘stroke patients’). However, BP control in stroke patients is known to be suboptimal^7^ and is estimated to increase the risk of recurrent stroke by 1/3 for every 10 mmHg increase in systolic BP.^8^

BP control is closely linked to BP management, which can often be suboptimal due to inertia from both patients and clinicians.^9^, ^10^, ^11^ In the UK and Western Europe, only around 14%‐26% of patients with uncontrolled hypertension have their medication increased.^12^ Any intervention hoping to improve BP management in stroke patients therefore needs to understand and address both patient‐ and clinician‐level barriers.

### 1.1 Developing interventions

The Medical Research Council (MRC) guidance on developing and evaluating complex interventions helped to standardize some key elements involved in intervention development^13^ such as identifying the evidence base and developing a strong and appropriate theoretical underpinning for an intervention. Paying attention to *context* is key: what works in one setting for one subgroup of the population may be less effective, or even harmful, elsewhere for other subgroups. Contextual factors require sensitive examination, not only when evaluating and implementing an intervention into the ‘real world’ but also from its inception.^14^ Newer guidance, specifically for intervention development,^15^ recognizes that context significantly impacts the appropriateness and success of any intervention, emphasizes the importance of involving stakeholders throughout development and recommends refining the intervention in response to stakeholder feedback.

Working with the target population during intervention development enables a detailed understanding of users’ psychosocial contexts, and the identification of potential barriers to the target behaviours.^15^ The person‐based approach (PBA)^16^ provides a clear process for achieving this. This iterative approach moves cyclically between data collection involving in‐depth qualitative research with the target user population, analysis to identify behavioural barriers, modifications to the intervention and then further data collection.^17^ Ideally, participants should be sampled carefully to reflect the diversity of people affected by the particular health condition. In reality, however, some groups are consistently under‐represented in research,^18^, ^19^, ^20^ which can lead to their perspectives being overlooked and their needs not addressed.^21^, ^22^

Another complementary approach to prioritizing users’ needs is to work closely with patient and public involvement (PPI) contributors. This enables members of the public to be actively involved in research,^23^ ideally at every stage of the research cycle.^24^ Although PPI does not provide ‘data’ like the qualitative interviews using PBA, PPI contributors provide valuable oversight on the research questions, design, methods, conduct and even interpretation of findings, ensuring these are in line with patient priorities and needs. Muller et al^25^ have highlighted the value of both PPI and PBA during all stages of intervention development. PPI enables a deep, longer‐term involvement of stakeholders in the conception, development and implementation of the intervention. Using PBA allows individuals who are not personally invested in the intervention to objectively inform its development from their varied perspectives and contexts.

### 1.2 Aim of this paper

This paper aims to describe the optimization of a digital intervention for stroke patients, particularly focusing on the value of involving a diverse range of participants, using the person‐based approach with integral patient and public involvement.

## 2 METHODS

This work was informed by the MRC guidance^13^ and a more up‐to‐date framework of actions for intervention development.^15^ In the early stages of planning, the study team reviewed published research from other BP self‐monitoring trials^26^, ^27^, ^28^ and drew on existing theories^29^ to inform the protocol and the logic model (Appendix S1). This paper describes the qualitative research and PPI that informed the development of the BP:Together intervention.

### 2.1 Design

**FIGURE 1:**
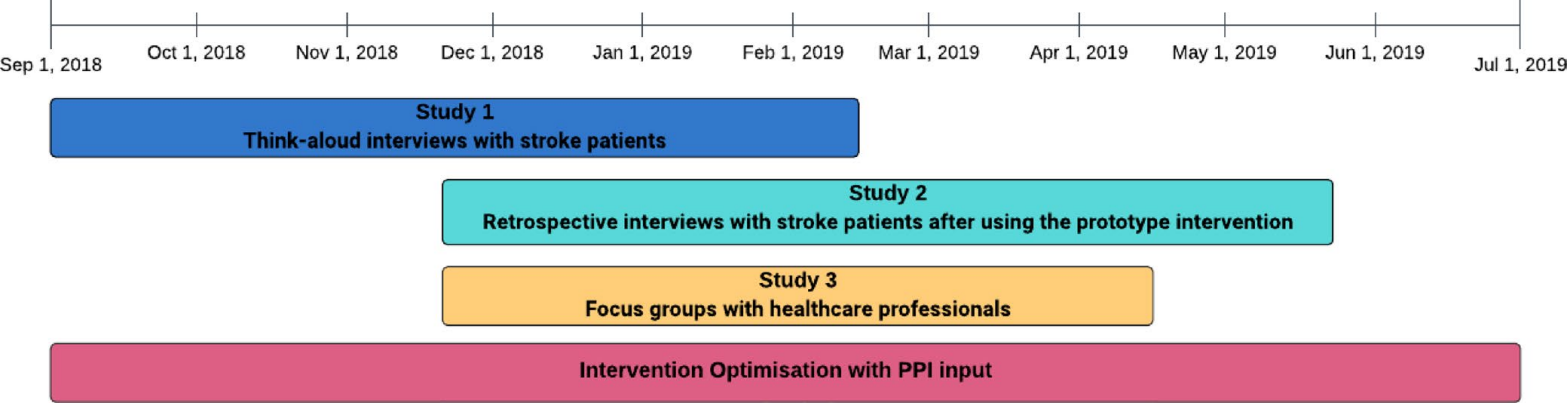
BP:Timeline for the three studies during intervention development

Researchers conducted three qualitative studies (Figure 1), using PBA methods with patients and health‐care professionals alongside on‐going PPI (see Section 2.3):

1. Study 1: Think‐aloud, face‐to‐face interviews^30^ with stroke patients (alone or with a family member) to explore perceptions of prototype intervention materials, identify possible barriers to engagement and modify as appropriate;
2. Study 2: Retrospective interviews with stroke patients (telephone or face‐to‐face, according to patient preference) who had used the prototype intervention for a week, to explore their experiences and identify further barriers to engagement;
3. Study 3: Focus groups with clinicians and support staff to explore feasibility and concerns about implementing the intervention in primary care.

Studies 1 and 3 were conducted in London and the Thames Valley area in England, and in the Mid‐west and Midlands in Ireland. Study 2 was only conducted in England as the technical systems supporting the prototype intervention were not in place for Ireland.

### 2.2 The BP:Together intervention

**FIGURE 2:**
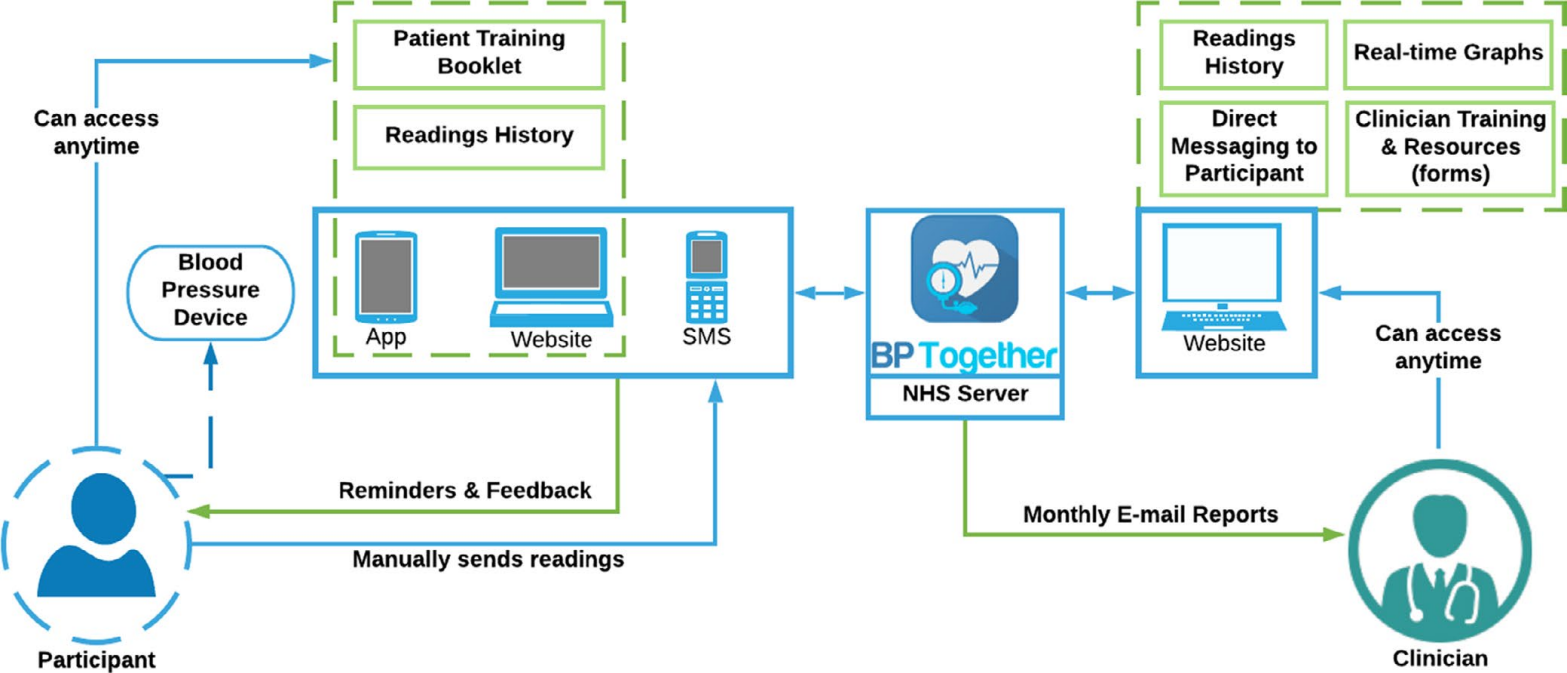
BP:Together system overview and interaction between intervention modules

The BP:Together intervention was adapted from the TASMINH4^28^ and HOME BP^17^ interventions but with design features and content specifically to facilitate its use following stroke/TIA. It supports self‐monitoring of blood pressure (BP) and enables patients to share BP readings taken at home with their clinician for the initiation of planned medication changes when average readings are above target. Figure 2 shows the system overview and interactions between the intervention modules.

When BP:Together is trialled in primary care, clinicians will complete a mandatory online training module about the intervention before recruiting any patients. Patients randomized to the intervention will receive in‐person training from a nurse and an information booklet (the ‘BP:Together booklet’) for on‐going support at home. The training session includes registering the patient on the digital intervention, the patient practising taking their own BP readings, and sending them via the website, SMS message or app, according to patient preference. The rationale for providing the intervention across three different interfaces is explained below:

1. The website enables access to the intervention via a desktop or laptop computer, which has a larger display and easier navigation via a mouse and keyboard, compared with using a smartphone or tablet.
2. For people who use smartphones or tablets, the app option provides an immersive experience allowing quick and easy access to information, notifications and BP records (graphs, filtered message history and help pages).
3. The SMS option does not require the use of a smartphone and is likely to be appreciated for its simplicity. It is ideal for participants who might be put off accessing the intervention if they had to use a smartphone or tablet. The SMS approach offers the essential basic functionality needed to interact with the intervention: the patient sends their readings in a toll‐free SMS message and receives SMS reminders and feedback on their readings each time.

Once the patient has been trained, the clinician reviews the patient's current antihypertensive medication and makes a three‐step plan for dose increases or additional drugs, to be actioned during the 12‐month programme.

The patient then receives reminder prompts in the format of their choice (email, app notification or SMS message) to measure their BP, and receives instant acknowledgements when readings are sent. The patient receives overall feedback based on their average, using an algorithm based on the NICE guidelines for stroke/TIA patients.^6^ If their average BP is above target (systolic BP above 125 mmHg), an email prompts the clinician to initiate the next medication change in their three‐step plan.^31^ In anticipation of clinical inertia arising from clinicians’ concerns that patients might not accept increases to their medication,^10^, ^32^ the BP:Together intervention also invites patients to send a pre‐set message to their clinician to confirm they are ready for the next planned medication change when their average reading is above target.

### 2.3 Patient and Public Involvement

Two *PPI co‐investigators* were recruited at the outset as part of the core research team for BP:Together. Both had previously had a stroke and had experience of self‐monitoring their BP. They helped write the protocol, attended all study team meetings and contributed to discussions regarding intervention procedures, content, qualitative study materials and data interpretation. One PPI co‐investigator also recruited participants from her local Stroke Association group.

Additionally, a senior representative of Speakeasy (a charity supporting people with aphasia) helped to develop aphasia‐friendly videos, information sheets and BP:Together booklets.

In the early planning stages (prior to studies 1, 2 and 3), two community groups also contributed to PPI activities (*community PPI*): Different Strokes Southampton and the Oxford Aphasia Group. Researchers (TR, CS or KM) visited each group three times to explore their beliefs about BP after a stroke, and gain feedback on the BP:Together procedures for medication change and accessibility of the aphasia‐friendly materials.

While developing the clinician training materials, researchers also consulted two research nurses with experience of conducting intervention research in primary care to ensure the training was clear and the procedures were feasible in practice.

### 2.4 Recruitment and procedure

Interviews and focus groups were conducted in phases with iterative amendments to the intervention based on on‐going analysis of feedback. Participants provided informed consent, and all interviews and focus groups were audio‐recorded and transcribed verbatim. During studies 1 and 2, if family members expressed interest in the interview/training session alongside the main participant (i.e. stroke patient), they were invited to join after signing a separate consent form. Extra time was scheduled for sessions with aphasic patients, especially if they had nobody at home to help them. Detailed field notes were taken after each interview. Recruitment ceased when the analysis suggested that data saturation^33^ was reached in feedback on the main components of the intervention. For intervention development, this means reaching a point whereby further data collection is unlikely to identify any new changes to the intervention.^17^

#### 2.4.1 Studies 1 and 2: Think‐aloud and retrospective interviews with stroke patients

Two recruitment approaches were used:

##### GP practices

Used search criteria to identify eligible patients based on history of stroke or TIA, and having a recent clinic reading of systolic BP above 130 mmHg. These patients received an invitation letter, a participant information sheet and a reply slip.

##### Community settings

To ensure inclusion of Black and minority ethnic (BAME) patients and those living in economically deprived areas, participants were also recruited from multiple community‐level sources. TR built relationships with the leaders of local churches and mosques whose congregations included BAME communities, and presented the research during their prayer services. A formal recruitment request sent to community‐based staff from the Stroke Association, and attendance of two stroke support group meetings in economically deprived neighbourhoods in Oxfordshire and London further aided recruitment. PPI co‐investigator MW visited the London group, enabling patient‐to‐patient recruitment.

Sampling of stroke patients was purposive based on age, gender, time since stroke/TIA and severity of post‐stroke/TIA impairment in terms of understanding written information, speaking, vision, and arm and head movement. Participants reported these characteristics on reply slips returned to the team.

For study 1, face‐to‐face think‐aloud interviews were conducted by TR in England and RD in Ireland. The interviews began by inductively exploring experiences of managing health after a stroke/TIA, and perceptions about BP. Participants then viewed the prototype BP:Together booklet, the website and/or the app with the researcher present, while saying out loud what they were thinking, thus sharing how they perceived it and potential barriers to engagement.^17^

For study 2, emulating the upcoming randomized controlled trial (RCT), TR gave participants in‐person training (Section 2.2) at their home and left them with a BP monitor, the BP:Together booklet and a diary to note any thoughts or feedback while self‐monitoring at home (to help with their recall during the interview). Participants then used the intervention to self‐monitor their BP at home over one week, and submitted their readings via SMS message, app or website. Retrospective, semi‐structured telephone interviews then explored participants’ experiences of using the intervention, specifically: self‐monitoring their BP; submitting their readings using the BP:Together programme; and receiving feedback on their BP readings from the programme. Telephone interviews were offered because the researcher had already visited the participant during the training session and established rapport with them, and a telephone call could be both practical and convenient for gathering the data needed. Participants were offered the option of a second face‐to‐face visit for the interview if they preferred.

During development, the intervention was not ‘live’: communication regarding participants’ BP readings was linked only to the study team and not to primary care.

#### 2.4.2 Study 3: Focus groups with health‐care professionals

Invitations were sent to GP practices in the study area. Clinicians and support staff (GPs, nurse prescribers, practice or research nurses, administrative staff, health‐care assistants) were invited by email (with an attached participant information sheet) from practices that expressed interest. We sought to gain insight into the process of implementing the intervention in primary care, from both the perspectives of clinicians and practice administrative staff who would provide practical support in processing the communication between patients and their GPs. Prior to the focus group, clinicians were asked to complete the online training module and record any thoughts to share. One or two facilitators were present at each focus group (KM, TR, CS or RD). During the focus group, open‐ended questions explored what participants liked and disliked about the intervention and training, and any facilitators or barriers to implementing it in practice.

### 2.5 Data analysis

Using the PBA approach,^16^, ^17^ researchers moved cyclically between data collection, analysis (to identify potential changes to the intervention), modifications to the intervention and then further data collection. KM and TR familiarized themselves with the interview recordings and transcripts. Quotes from the transcripts were extracted and recorded in a ‘table of changes’ according to the relevant section of the intervention and whether they were positive or negative. This was a highly rigorous process, which involved rapidly collating *all* feedback relating to each aspect of the intervention, to facilitate discussion of potential iterative changes, drawing on evidence, theory, and PPI and expert opinion. Appropriate modifications were implemented by KM and CrR when deemed important to overcome barriers to engagement, thus making the intervention more acceptable, persuasive and feasible to implement. A set of criteria^34^ were used to record the reason for and importance of making the change.

## 3 FINDINGS

### Participants

#### Study 1

29 think‐aloud interviews (24 in England, 5 in Ireland; Table 1) of which 11 also included a partner or other family member involved in the day‐to‐day care of the study participant. Participant names are pseudonyms.

**TABLE 1:** Patient characteristics for Study 1 (England and Ireland, n = 29) and Study 2 (England, n = 11)

Gender	
Male	24
Female	16
Age	
35‐50	4
51‐60	5
61‐70	13
71‐80	11
>80	7
Recruitment via	
Primary care	27
Community	13

#### Study 2

11 semi‐structured telephone interviews in England with participants who had used the intervention (Table 1), either independently or with the help of a family member. Five participants used SMS messages to send their readings (with optional access to the BP:Together website), one used the BP:Together website only, and five used the BP:Together app downloaded onto their tablet or smartphones.

#### Study 3

Seven focus groups with a total of 25 GPs, 3 practice nurses, 2 nurse prescribers, 2 research nurses, 2 members of the administration team and 1 health‐care assistant.

The behavioural barriers identified from the qualitative data and PPI feedback, and the solutions used to address these in BP:Together are listed in Table 2. The four key recurrent barriers are discussed in more detail below, to demonstrate how optimization of the intervention was enhanced by seeking feedback from a diverse range of sources.

**TABLE 2:** The behavioural barriers that were identified from the qualitative data and PPI feedback, and the solutions used to address these in BP:Together

Behavioural barrier	Raised by	Quote	Solution
Intervention materials may be burdensome and inaccessible for people with aphasia	PBA interviews, PPI and stakeholders	I: How do you find it [the booklet]? R: More confusing. I: Tell me what's confusing cos we need to know. R: To retain in my head what I’m reading is impossible…. You can change when, when you take your blood pressure reading over the week to fit your, your time. Here is an example, do I have to read all of this? (Matthew, 76 years)	Introduced essential face‐to‐face training, and a short summary sheet for recording BP readings, which included the key information about how to send them. Optional video available for people to see how to take a reading. Adjusted booklet to adhere to guidance from NIHR for aphasia, and sought input from a senior representative of Speakeasy (a charity supporting people with aphasia)
Need for support from others to take blood pressure and/or send readings	PBA interviews and PPI	[re putting BP monitor cuff round arm] This is what I would do, yeah. There's no way he can do it himself. (Margaret, partner of John, 63 years)	Ensured that intervention was designed for family members to be involved.
Lower health literacy leading to lack of motivation to engage in BP self‐monitoring	PBA interviews	I’m not going to have another stroke, Jane. Seven years on, no that's not happening in my view. […] Well that's how I feel…So I don't think it's going to happen to me, again. Next! (Carl, 58 years)	Clearer explanation of the purpose of the intervention and the benefit of controlling BP for future health. Added to clinician training an explanation of some of the common misconceptions patients might hold about stroke, and encouraged them to address these at the baseline review with the patient.
Belief that changing medication should be the responsibility of the GP alone, not for the patient to decide	PBA interviews, PPI	There's too, there's too much power being given to the person is the patient. All the power and decisions have to be the GP's (Peter, 70 years)	Careful adjustment to the message for patients to agree to a pre‐planned medication change when their readings are too high, as well as clear rationale for this in the booklet.
Concern that the intervention places additional burden on GPs	PBA interviews	Putting more pressure on the doctors, I’m not that happy with that because they've got enough to do already and if you pile too much pressure on them then it just causes more problems. (Carl, 58 years)	Added an explanation to the BP:Together booklet that the intervention helps GPs to understand your blood pressure. Discussion at the baseline medication review will also help reassure patients that their GP is committed to the intervention.
Confusion over the purpose of the intervention	PBA interviews	What, what's the, the, the point? To take more care of [er] the patient? (Joe, 76 years)	Added a clear explanation at the start of the BP:Together booklet that BP:Together can help you and your GP to keep an eye on your BP and find the right medicine for you. This helps reduce the risk of another stroke or TIA.
Low confidence that BP target would be achievable.	PBA interviews	Patient: Well that's very rare below 125. I have it below 125 but… Family member: But it's not often, that's right. (Stephen, 80 years)	Modified the training for GPs re the baseline medication review to normalize the BP targets for the patient and reassure them that it is OK to exceed the targets at first.
Confusion over the instructions for sending BP readings by SMS	PBA interviews	I’m just wondering, that's a very long number, so 134 is my blood pressure result, 84 then is my second one and 127 74, I’m not too sure what that is. (Siobhan, 82 years, Ireland)	Changed the BP:Together booklet to include images of the BP monitor and the SMS message with clear step‐by‐step instructions for how to send two readings. Ensured that each participant would receive face‐to‐face training on how to send BP readings.
Concern about the feasibility of taking readings at regular times	PBA interviews, PPI	I think to have, to have to do it in the morning, to have to do it in the afternoon, is probably, would be hard work because you don't know what time you're going to wake up. You don't know how you're going to feel when you wake up and you don't know what's going on in your life. (Carl, 58 years)	Changed the BP:Together booklet to emphasize the flexibility of the BP monitoring process. Introduced a case study example of a participant, who missed her readings on some days and changed the time of day to suit her routine.
Anxiety about being told average BP is above target, and having to wait to take more readings	PBA interviews	I just think I would be anxious if mine was yellow and I’d have to wait to do mine again the next week. I’m not saying you'd have a stroke in the week but I’d just be concerned and worried about it if they weren't right. (Anna, 58 years, Ireland)	Adjusted the BP:Together booklet and the feedback message received by patients at the time of submitting their readings to reassure them that their BP is only slightly above target
Concern about increased workload if patients are anxious about their home readings	HCP focus groups	There might be somebody genuinely worried with the 150 over 90, wondering should I be doing something now, but in terms of response time, time to get the feedback, that could cause some anxiety, they might be contacting the GP again wondering can you do anything. (GP, Ireland focus group 3)	Added to online clinician training that patients receive instant feedback on their readings from the intervention with clear directions for when to take urgent action
Concern about changing medication without understanding patients' symptoms	HCP focus groups	The patients are texting their digits but this is not taking in to account their symptoms. We are not getting any idea what their symptoms are. (GP, Ireland focus group 3)	Patients are asked to message the GP to indicate if they feel ready for the next medication change to be made, which could help reassure clinicians. The intervention encourages patients to make an appointment to see the GP when needed.
Stroke patients may struggle to remember conversations with GP by telephone	Study team	N/A	Added to online clinician training and emails for GPs notifying re medication change that following up a phone call with a written message may be helpful.
Concerns about the workload of monitoring a patient message system	HCP focus groups	We've got a 150 people thinking oh I think it's a great idea to let Dr xxxxx know about my issues and he's got a busy workload, home visits to do plus 40 messages from the patients and one of them saying I want to kill myself. (GP, UK focus group 1)	Removed the option for open‐text messaging from the patient to the GP due to the issues of safety in continually monitoring this. Instead, offered patients a 'call me' system whereby they can request a call‐back.
Concerns about patient becoming hypotensive if you change their medication	HCP focus groups	The fear of causing problems for a small few stop us from reducing the blood pressure for a larger group. And I think that's the biggest problem, the fear is you don't know who is going to get the problem. Therefore, with a wider group you tend to be more conservative. (GP, Ireland focus group 1)	Addition of evidence to clinician online training to show that previous interventions did not lead to more side effects, and clarification that people with postural hypertension will not be eligible.

### 3.1 Intervention materials for people with aphasia

Heeding early advice from our PPI co‐investigators and community PPI sessions to accommodate stroke patients with aphasia, the study team initially developed two versions of the BP:Together booklet: a standard version and an aphasia‐friendly version, as per NIHR guidelines.^35^ Despite including less text, the aphasia‐friendly version was much longer than the standard version as it included a picture to demonstrate each point and more spaced‐out text. Our PPI contributors felt this was problematic as it seemed too long to be engaging and accessible.

However, the first few think‐aloud interviews indicated that some participants who had not self‐identified as having aphasia, and hence received the standard version of the BP:Together booklet, struggled to read it. Carl, who is White and lives in a council estate with his partner, said:

You can read it [speaking to partner]. Oh, you know, it takes me for…I’m trying to read. It’s hard enough for me to live. I struggle right, it takes me ages to read things. She just goes, wallop, wallop, wallop. She’s really good at it. (Carl, 58 years)

**FIGURE 3:**
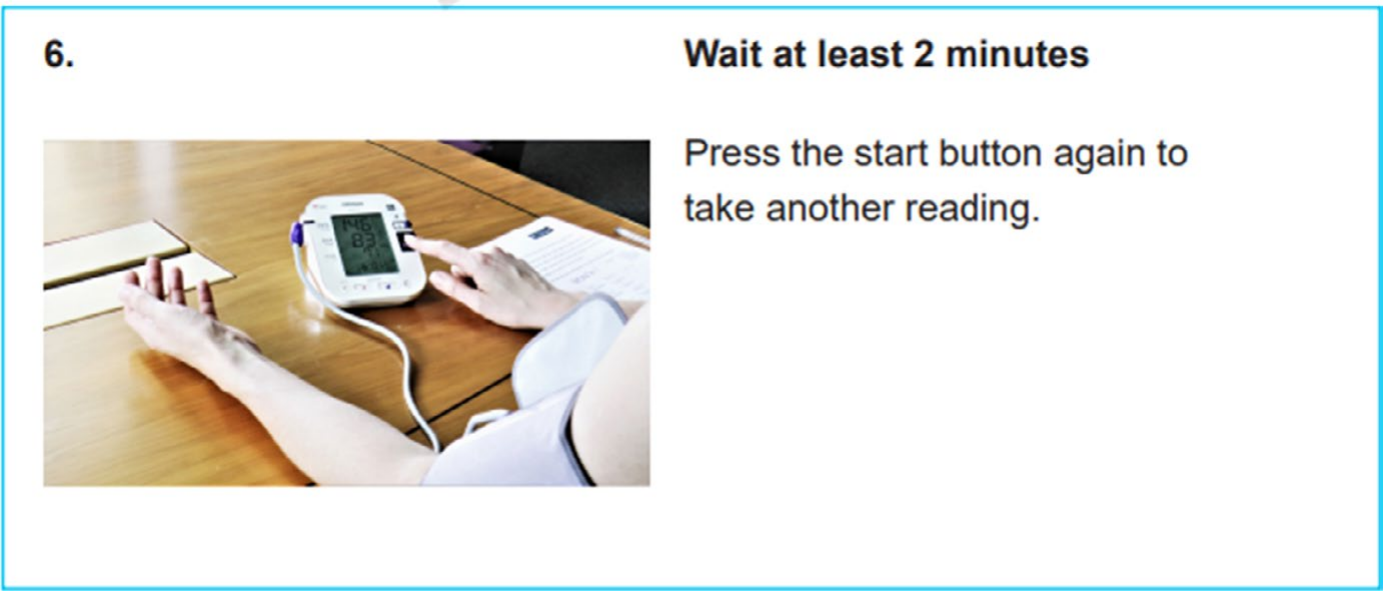
Example of aphasia‐friendly guidance from the BP:Together booklet

This feedback had important implications for accessibility of the intervention. Not wanting to exclude potential users from socially or educationally compromised backgrounds, it was agreed with the wider trial research team to have only *one* version of all the study materials that was as aphasia‐friendly as possible. The rest of the think‐aloud interviews were used to learn how best to rationalize the content to convey all the necessary information, supported by images where possible (eg Figure 3), without being over‐long or difficult to navigate.

Later think‐aloud interviews suggested that stroke patients found the updated BP:Together booklet easy to follow. During the retrospective interviews in Study 2, a few participants admitted they rarely referred to the booklet; only consulting it if there was a problem, such as an error on the BP monitor. Such feedback highlighted the importance of the one‐to‐one training session at the start of the intervention to promote understanding. It also prompted us to include a short, image‐based reminder of how to take and send a BP reading with the monitoring record sheets, thus ensuring participants had instructions to hand regardless of whether they consulted the BP:Together booklet. An aphasia‐friendly video explaining the participant information sheet was also developed to facilitate recruitment to the RCT.

### 3.2 Need for additional support

Participants’ family members often acted as their carers and advocates, especially when the stroke had caused communication or mobility problems. This was an issue anticipated by our PPI co‐investigators. Lily had moderate aphasia and struggled to say more than a few words at a time. Her husband Thomas described how he helped her with her BP management:

Interviewer: […] do you look at your blood pressure, either at the GP’s or at home?

Thomas: We usually, like the flu jab and that sort of thing…

Lily: Yes.

Thomas: Then I ask her [the nurse] just to check the blood pressure. I don’t know if they would do it automatically but….

Lily: Yes, yes, yes.

Thomas: So we go down there. It’s mainly by request really… and if [Lily’s] not feeling particularly well, I’ll monitor the blood pressure at home for a few days. (Thomas, partner of Lily, 70 years)

There were also occasions where the stroke patient was less interested in learning about the intervention than other family members. Habib, an elderly, Asian man who had recently suffered a second stroke lives with his wife and their son's family, and his daughter lives nearby. He was not very aware of his health status so his daughter‐in‐law, Shehla joined the interview. Shehla was able to discuss Habib's health‐related behaviours, including BP measurement, and give detailed feedback on the study materials. Here, she describes how BP:Together could help the family manage Habib's BP better, in the context of his other comorbidities:

We don’t know when it’s properly controlled with the medication, because he does feel up and down. But then obviously with this programme he will have the insurance that okay, we give the readings, we know where he's standing, the medication is working and he’s fine in that sense. And all we have to concentrate on is the diabetes. (Shehla, daughter‐in‐law of Habib, 76 years)

Shehla warned that if Habib were to do the intervention, his BP readings would need to be sent via her or her husband's phone, as Habib rarely used his mobile phone. This was also the case for Sam, a participant in the retrospective interview study. Although Sam took his readings himself, he asked his son Oscar, who lived next door, to download the BP:Together app on his (Oscar's) phone, and would communicate his readings to Oscar each time to send across. These intergenerational supportive arrangements were more prominent among participants recruited from community settings.

This phase of work highlighted the extent to which family members’ lives had been affected by their relative's stroke. Many were deeply involved in the day‐to‐day care of the person living with stroke/TIA; therefore, successful participation in any intervention would require engendering trust and buy‐in from these significant others and the primary participant. Developing a separate BP:Together booklet for family members was considered by the study team, but the interviews suggested they liked the patient booklet. Although BP:Together only registers one telephone number per participant, the study team accepted that sometimes the person communicating with the programme (and registering their digital device) may be another household member rather than the patient themselves. Recognizing the role families play in the care of stroke patients may allow inclusion of participants who lack basic digital skills, but will still be able to use and benefit from the intervention.

### 3.3 Lower health literacy affecting motivation

Deliberate sampling for a socio‐economically and ethnically diverse sample reflective of stroke demographics in the population generated rich data, in terms of both how the stroke had affected different individuals and how they approached the BP:Together intervention. Participants recruited via GP practices appeared more engaged with their health and were already undertaking behaviours to reduce their risk of another cardiovascular event. In contrast, community‐recruited participants seemed to see their GP less often, showed lower levels of health literacy and were less enthusiastic about seeking medical advice or adopting behaviours to promote better health. Some had significant comorbidities, including poor mental health, and the stroke had severely disrupted their lives. David is 62 years old, Black and severely aphasic following a stroke in 2015. He lives alone in a sheltered council flat. A carer visits him 3 days a week. His daughter and grandchild live in another city and visit him twice a month.

Interviewer: […] and how do you feel about, about blood pressure? You said you think it’s high.

David: To be honest, I don’t care. I’m old enough now, my daughter is happy that’s all.

Interviewer: [laughs] You’re old enough now. What does that mean?

David:I’m over sixty and I just think I could fall now, I don’t care.

Interviewer: You don’t care about your health.

David: [Nods to indicate ‘no’] (David, 62 years)

Alongside low levels of engagement with their health, knowledge around BP and stroke also varied, with many being unaware of the link between BP and stroke risk. The think‐aloud interviews allowed the study team to explore how different people viewed their health risks in general, and some of the beliefs these were based on. Carl (mentioned previously) saw stroke as a one‐time event, so having had his, he felt he was not at risk:

Carl: [talking to his partner, Jane] I’m not going to have another stroke, Jane. Seven years on, no that’s not happening in my view. […] Well that’s how I feel…So I don’t think it’s going to happen to me, again. Next!

The clinician online training was optimized to identify some of the common misconceptions patients hold about BP and stroke based on these insights into low motivation and health literacy, and trains clinicians to discuss these openly with patients at the start. Also, the BP:Together booklet was optimized to include a clearer explanation of the rationale for the intervention, emphasizing the value of controlling BP to avoid a subsequent stroke.

### 3.4 Beliefs about changing medication

In a community PPI session, stroke patients and family members expressed preference for the medication change process to be initiated by the clinician, with no input from the patient. They felt placing responsibility onto the patient to act could be challenging for people with cognitive or memory impairments. Similarly, participants in the early think‐aloud interviews were uncomfortable about BP:Together prompting them to contact their clinician when their readings were raised.

here it’s almost sounding like, we’re telling the GP, this is what you have to do.…. I don’t see why the patient should be asking the GP to change their medication. (Lara, 42 years)

The doctor will know the result, he’ll know whether the medicine needs changing, so it’s a bit odd that [patient name] will take charge of it if he needs the next medicine in his plan. (Sarah, partner of Philip, 53 years)

Patient‐initiated medication change has recognized benefits in terms of overcoming clinical inertia.^31^, ^36^ Evidence suggests clinicians can be reluctant to increase antihypertensive medication,^32^, ^37^ whereas patients can feel empowered by being involved in their medication change and confident to request their next medication change when needed.^38^ Working closely with the PPI co‐investigators, the researchers carefully navigated their way through the stated reluctance from participants, trying out multiple iterations of the particular phrasing of the feedback message that invited them to contact the clinician following raised readings. For instance, asking patients to send a message saying ‘I’m ready’ rather than ‘Change’ (following an average reading recommending a medication increase) was an important distinction which patients were much happier with, as it avoided implying they were instructing the clinician, and instead simply communicated to their clinician that they were comfortable and ready to make the medication change.

The BP:Together booklet was also adjusted to clearly explain to patients the rationale for contacting the clinician about medication changes, including a fictional quote from a ‘busy GP’ explaining how receiving such a message helped her.

Clinicians in the focus groups felt reassured by patient involvement in the medication change process. Some felt that patients may not be willing to change their medication if it was purely clinician‐driven.

You’d have to pick your patient. It’s my experience that they don’t take the tablets that you give them, never mind increase them. (Health‐care professional focus group 4)

In response to these concerns, the baseline medication review in which the clinician makes the three‐step plan with the patient was modified to include a guided discussion to manage both patients’ and clinicians’ expectations about medication change, reassuring each party that the other was happy with the process.

## 4 DISCUSSION

### 4.1 Summary of findings

This paper illustrates the value of using the person‐based approach during intervention development, and the importance of seeking a diverse sample from the target population. The visual format and language used in all BP:Together materials was tailored to accommodate heterogeneity within the stroke population in terms of language ability, demographics, and digital and health literacy. Household members can support stroke patients to use BP:Together, especially those with cognitive or physical impairments. BP:Together was also feasible to implement in primary care as part of a planned trial (https://doi.org/10.1186/ISRCTN57946500).

The qualitative PBA research provided in‐depth insights into important behavioural barriers that the intervention needed to address, such as patients feeling helpless about their own ability to alter their risk of a future stroke, or feeling anxious about medication changes. PPI provided essential contribution into the design of the intervention and research decisions from an early stage. Recognized as a familiar issue,^25^ the PPI contributors did not adequately represent the diversity of the stroke‐affected population. Extra efforts to recruit a wide variety of participants in terms of socio‐demographics and post‐stroke impairments provided these multiple perspectives. Meanwhile having PPI input throughout, including as co‐investigators, ensured that the ‘patient voice’ was present in all decision‐making. Both activities helped optimize the intervention by checking that the materials were understandable, convincing and feasible for integrating into everyday life, and that possible setbacks to successful implementation were addressed.^25^ Both PPI and PBA drew on core values and aspirations for working with the public including ‘genuine involvement; a focus on understanding many perspectives; an iterative and investigatory style; and a commitment to changing things in the interest of those who have to live and work with the results’.^39^

### 4.2 Aphasia and digital literacy can take many forms

By deliberately sampling to include participants who are typically under‐represented in research, researchers were able to explore the ways in which health interventions can unintentionally become exclusionary. The study team had initially assumed that only people who self‐identified as aphasic needed the simplified, text‐light and picture‐based version of the study information. However, through talking to people with lower literacy levels the researchers realized that this two‐tier approach would render the intervention inaccessible to many, when in fact, simpler, easily accessible interventions appeal to most people.^40^ Given the correlation between social disadvantage and poor health,^41^ neglecting such an issue runs the risk of exacerbating existing health inequities. This is particularly important in the case of digital health interventions, which are often hailed as having the potential to democratize health care by empowering everybody irrespective of social status, and levelling out long‐standing social gradients in health.^42^, ^43^, ^44^ Although this position has increasingly been critiqued,^45^, ^46^, ^47^ it is an attractive hypothesis, strengthened by data showing high levels of ownership of mobile phones across young and old, and rich and poor,^48^ including those with lower literacy and/or limited English‐language skills. However, *access* to technology must not be mistaken for actual *use* of technology.^47^ Several participants when first contacted admitted to possessing a mobile phone or tablet, but at the interview, it would become apparent that they maintained a respectful but somewhat remote relationship with it.^49^, ^50^ At first, this appeared as quite a stubborn barrier to participation, but was quickly resolved with the realization that family members might take on a crucial role, thus allowing engagement via existing ‘chronic care infrastructures’ present at home.^51^, ^52^ Younger family members often tended to have higher health and technological literacy than the stroke patient and were already involved in their care. This was a way to avoid excluding this (potentially large) important group of intervention users.

### 4.3 Role of research in resisting existing racial and socio‐economic inequalities

Some demographic groups are too often labelled as ‘hard‐to‐reach’ in research practice. However, they *are* accessible if research teams engage with those communities and build relationships in meaningful ways.^53^, ^54^ Evidence suggests a chronic under‐representation of people from BAME and low socio‐economic groups in medical research, including in cardiovascular trials.^18^, ^19^, ^20^, ^55^ This is particularly significant for stroke research as BAME groups and those residing in high‐deprivation areas have a higher incidence, earlier age of onset and greater stroke severity compared with other groups.^2^, ^3^, ^4^ Digital health interventions cannot hope to become equitable without first considering existing patterns of public engagement with digital health, and the ways in which these are determined by factors such as education, age, computer skills, ethnicity and economic status.^56^ Almost all study participants recruited via community settings had never participated in research before but were enthusiastic when approached in person with a simple and friendly explanation about the purpose of the research, the opportunity to ask questions and have their BP measured. While some found it surprising and unfamiliar being invited to voice their opinions, once the researcher had built some rapport with them, they felt more comfortable about sharing their thoughts.

### 4.4 Strengths and limitations

The main findings in this paper offer important insights that go beyond this particular case and might be useful to other researchers developing complex health interventions. The main strength of this intervention development work was to combine different approaches to gain insights into the multiple landscapes of ‘life after stroke’. This multi‐pronged approach guided the team in shaping how the intervention could be incorporated into people's lives, while acknowledging that some barriers were immutable (eg the participant or household member needs to have a mobile phone). Although resource and time constraints limited this study to a small set of geographical locations, community‐based recruitment from poorer neighbourhoods in the Thames Valley and London ensured wide diversity across ethnicity and socio‐economic status. The project timelines also limited the number of participants interviewed, which may mean that some outstanding barriers to behaviour change were not addressed.

### 4.5 Conclusions

In conclusion, thinking about how an intervention will work must begin by examining the factors that produce and sustain poor health in the first place.^14^ Health problems that interventions seek to ameliorate should be explored in the social context in which they exist,^15^ with an understanding of the mechanisms that render some people at greater risk than others. Any planned intervention must make clear how it anticipates responding to those problems.^14^ Ultimately, the aim of public health practice is to improve health for all, especially those who have the greatest potential margin of benefit from the intervention. This work demonstrating the integration of the person‐based approach and PPI will hopefully inform wider discussions regarding the development of complex health interventions to make them attractive, engaging, useable and most importantly beneficial, for the widest range of people.

## CONFLICT OF INTEREST

Richard McManus has received BP monitors for research from Omron and is working with them on the development of a telemonitoring system. There are no other conflicts of interest.

## AUTHORS’ CONTRIBUTIONS

RJM, LH, LY, LT and MW were involved in conception of the project, developing the proposal, study design and securing funding. TR, RD, KM and CS conducted the data collection. KM and TR analysed the interview data, and sought regular input from CaR and MW to interpret data and make decisions about optimizations. CrR designed and developed the software intervention. CV designed the system. CV and LT guided the engineering development. RJM made clinical decisions about the intervention and optimizations. TR drafted the article. All authors were involved in planning and developing the intervention and decisions about the intervention content, contributed to critical revision of the article and gave approval of the final version to be published. LH is the guarantor for this work.

## ETHICAL APPROVAL

Ethics reviews were carried out by the Proportionate Review Sub‐committee of the HSC REC B (REC reference: 18/NI/0134) and University Hospital Limerick Ethics Committee (Ref 077/19).

## Supporting information

Appendix S1

## Data Availability

The data that support the findings of this study are available on request from the corresponding author. The data are not publicly available due to privacy or ethical restrictions.

## References

[1] Stroke Association . State of the nation: Stroke statistics February. London, UK: Stroke Association; 2018.

[2] Wang Y , Rudd AG , Wolfe CD . Age and ethnic disparities in incidence of stroke over time: the South London Stroke Register. Stroke. 2013;44(12):3298‐3304. 24114452 10.1161/STROKEAHA.113.002604

[3] Marshall IJ , Wang Y , Crichton S , McKevitt C , Rudd AG , Wolfe CD . The effects of socioeconomic status on stroke risk and outcomes. Lancet Neurol. 2015;14(12):1206‐1218. 26581971 10.1016/S1474-4422(15)00200-8

[4] McFadden E , Luben R , Wareham N , Bingham S , Khaw KT . Occupational social class, risk factors and cardiovascular disease incidence in men and women: a prospective study in the European Prospective Investigation of Cancer and Nutrition in Norfolk (EPIC‐Norfolk) cohort. Eur J Epidemiol. 2008;23(7):449‐458. 18509727 10.1007/s10654-008-9262-2

[5] Katsanos AH , Filippatou A , Manios E , et al. Blood Pressure Reduction and Secondary Stroke Prevention: A Systematic Review and Metaregression Analysis of Randomized Clinical Trials. Hypertension. 2017;69(1):171‐179. 27802419 10.1161/HYPERTENSIONAHA.116.08485

[6] Intercollegiate Stroke Working Party . National Clinical Guideline for Stroke. 5th edn. London, UK: Royal College of Physicians; 2016.

[7] Falaschetti E , Mindell J , Knott C , Poulter N . Hypertension management in England: a serial cross‐sectional study from 1994 to 2011. Lancet. 2014;383(9932):1912‐1919. 24881995 10.1016/S0140-6736(14)60688-7

[8] Law MR , Morris JK , Wald NJ . Use of blood pressure lowering drugs in the prevention of cardiovascular disease: meta‐analysis of 147 randomised trials in the context of expectations from prospective epidemiological studies. BMJ. 2009;338:b1665. 19454737 10.1136/bmj.b1665 PMC2684577

[9] Jamison J , Graffy J , Mullis R , Mant J , Sutton S . Barriers to medication adherence for the secondary prevention of stroke: a qualitative interview study in primary care. Br J Gen Pract. 2016;66(649):e568‐e576. 27215572 10.3399/bjgp16X685609 PMC4979933

[10] Phillips LS , Branch WT , Cook CB , et al. Clinical inertia. Ann Intern Med. 2001;135(9):825‐834. 11694107 10.7326/0003-4819-135-9-200111060-00012

[11] Milman T , Joundi RA , Alotaibi NM , Saposnik G . Clinical inertia in the pharmacological management of hypertension: A systematic review and meta‐analysis. Medicine (Baltimore). 2018;97(25):e11121. 29924011 10.1097/MD.0000000000011121 PMC6025046

[12] Wang YR , Alexander GC , Stafford RS . Outpatient hypertension treatment, treatment intensification, and control in Western Europe and the United States. Arch Intern Med. 2007;167(2):141‐147. 17242314 10.1001/archinte.167.2.141

[13] Craig P , Dieppe P , Macintyre S , Michie S , Nazareth I , Petticrew M . Developing and evaluating complex interventions: the new Medical Research Council guidance. BMJ. 2008;337:a1655. 18824488 10.1136/bmj.a1655 PMC2769032

[14] Moore GF , Evans RE . What theory, for whom and in which context? Reflections on the application of theory in the development and evaluation of complex population health interventions. SSM ‐ Population Health. 2017;3:132‐135. 29302610 10.1016/j.ssmph.2016.12.005 PMC5742639

[15] Cathain A , Croot L , Duncan E , et al. Guidance on how to develop complex interventions to improve health and healthcare. BMJ Open. 2019;9(8):e029954. 10.1136/bmjopen-2019-029954 PMC6701588 31420394

[16] Yardley L , Morrison L , Bradbury K , Muller I . The person‐based approach to intervention development: application to digital health‐related behavior change interventions. J Med Internet Res. 2015;17(1):e30. 25639757 10.2196/jmir.4055 PMC4327440

[17] Bradbury K , Morton K , Band R , et al. Using the Person‐Based Approach to optimise a digital intervention for the management of hypertension. PLoS One. 2018;13(5):e0196868. 29723262 10.1371/journal.pone.0196868 PMC5933761

[18] Mason S , Hussain‐Gambles M , Leese B , Atkin K , Brown J . Representation of South Asian people in randomised clinical trials: analysis of trials' data. BMJ. 2003;326(7401):1244‐1245. 12791739 10.1136/bmj.326.7401.1244 PMC161554

[19] Magin P , Victoire A , Zhen XM , et al. Under‐reporting of socioeconomic status of patients in stroke trials: adherence to CONSORT principles. Stroke. 2013;44(10):2920‐2922. 23899911 10.1161/STROKEAHA.113.002414 PMC4273080

[20] Bartlett C , Davey P , Dieppe P , Doyal L , Ebrahim S , Egger M . Women, older persons, and ethnic minorities: factors associated with their inclusion in randomised trials of statins 1990 to 2001. Heart. 2003;89(3):327‐328. 12591845 10.1136/heart.89.3.327 PMC1767569

[21] Clark LT , Watkins L , Piña IL , et al. Increasing Diversity in Clinical Trials: Overcoming Critical Barriers. Curr Probl Cardiol. 2019;44(5):148‐172. 30545650 10.1016/j.cpcardiol.2018.11.002

[22] Stronks K , Wieringa NF , Hardon A . Confronting diversity in the production of clinical evidence goes beyond merely including under‐represented groups in clinical trials. Trials. 2013;14:177. 23768231 10.1186/1745-6215-14-177 PMC3689626

[23] Going The Extra Mile: Improving The Nation’s Health And Wellbeing Through Public Involvement In Research. NIHR. 2015. https://www.nihr.ac.uk/documents/about‐us/our‐contribution‐to‐research/how‐we‐involve‐patients‐carers‐and‐the‐public/Goingthe‐Extra‐Mile.pdf

[24] Day S , Blumberg M , Vu T , Zhao Y , Rennie S , Tucker JD . Stakeholder engagement to inform HIV clinical trials: a systematic review of the evidence. J Int AIDS Soc. 2018;21(Suppl 7):e25174. 30334358 10.1002/jia2.25174 PMC6192899

[25] Muller I , Santer M , Morrison L , et al. Combining qualitative research with PPI: reflections on using the person‐based approach for developing behavioural interventions. Res Invol Engage. 2019;5(1):34. 10.1186/s40900-019-0169-8 PMC6857167 31807316

[26] Band R , Morton K , Stuart B , et al. Home and Online Management and Evaluation of Blood Pressure (HOME BP) digital intervention for self‐management of uncontrolled, essential hypertension: a protocol for the randomised controlled HOME BP trial. BMJ Open. 2016;6(11):e012684. 10.1136/bmjopen-2016-012684 PMC5129001 27821598

[27] McKinstry B , Hanley J , Wild S , et al. Telemonitoring based service redesign for the management of uncontrolled hypertension: multicentre randomised controlled trial. Br Med J. 2013;346:f3030. 23709583 10.1136/bmj.f3030 PMC3663293

[28] McManus RJ , Mant J , Franssen M , et al. Efficacy of self‐monitored blood pressure, with or without telemonitoring, for titration of antihypertensive medication (TASMINH4): an unmasked randomised controlled trial. Lancet. 2018;391(10124):949‐959. 29499873 10.1016/S0140-6736(18)30309-X PMC5854463

[29] Atkins L , Francis J , Islam R , et al. A guide to using the Theoretical Domains Framework of behaviour change to investigate implementation problems. Implement Sci. 2017;12(1):77. 28637486 10.1186/s13012-017-0605-9 PMC5480145

[30] van den Haak MJ , de Jong MDT , Schellens PJ . Evaluation of an Informational Web Site: Three Variants of the Think‐aloud Method Compared. Tech Commun. 2007;54(1):58‐71.

[31] McManus RJ , Mant J , Haque MS , et al. Effect of self‐monitoring and medication self‐titration on systolic blood pressure in hypertensive patients at high risk of cardiovascular disease: the TASMIN‐SR randomized clinical trial. JAMA. 2014;312(8):799‐808. 25157723 10.1001/jama.2014.10057

[32] Khatib R , Schwalm JD , Yusuf S , et al. Patient and healthcare provider barriers to hypertension awareness, treatment and follow up: a systematic review and meta‐analysis of qualitative and quantitative studies. PLoS One. 2014;9(1):e84238. 24454721 10.1371/journal.pone.0084238 PMC3893097

[33] Silverman D . Qualitative Research. London: Sage; 2016.

[34] Bradbury K , Watts S , Arden‐Close E , Yardley L , Lewith G . Developing Digital Interventions: A Methodological Guide. Evid‐Based Complement Alternat Med. 2014;2014:561320. 24648848 10.1155/2014/561320 PMC3932254

[35] Stroke NIFHRCRN . Enabling People With Aphasia To Participate In Research: Resources For Stroke Researcher. National Institute for Health Research Clinical Research Network: Stroke;2014. https://www.nihr.ac.uk/explore‐nihr/specialties/stroke.htm

[36] Schwartz CL , Seyed‐Safi A , Haque S , et al. Do patients actually do what we ask: patient fidelity and persistence to the Targets and Self‐Management for the Control of Blood Pressure in Stroke and at Risk Groups blood pressure self‐management intervention. J Hypertens. 2018;36(8):1753‐1761. 29889157 10.1097/HJH.0000000000001738

[37] Jones MI , Greenfield SM , Bray EP , et al. Patient self‐monitoring of blood pressure and self‐titration of medication in primary care: the TASMINH2 trial qualitative study of health professionals' experiences. Br J Gen Pract. 2013;63(611):e378‐e385. 23735408 10.3399/bjgp13X668168 PMC3662454

[38] Jones MI , Greenfield SM , Bray EP , et al. Patients' experiences of self‐monitoring blood pressure and self‐titration of medication: the TASMINH2 trial qualitative study. Br J Gen Pract. 2012;62(595):e135‐e142. 22520791 10.3399/bjgp12X625201 PMC3268493

[39] Locock L , Boaz A . Drawing straight lines along blurred boundaries: qualitative research, patient and public involvement in medical research, co‐production and co‐design. Evid Policy J Res, Debate Pract. 2019;15(3):409‐421.

[40] Rowsell A , Muller I , Murray E , et al. Views of People With High and Low Levels of Health Literacy About a Digital Intervention to Promote Physical Activity for Diabetes: A Qualitative Study in Five Countries. J Med Internet Res. 2015;17(10):e230. 26459743 10.2196/jmir.4999 PMC4642371

[41] Marmot M , Allen J , Boyce T , Goldblatt P , Morrison J . Health Equity in England: The Marmot Review 10 Years On. London, UK: The Institute of Health Equity; 2020. https://www.health.org.uk/publications/reports/the‐marmot‐review‐10‐years‐on

[42] Department of Health and Social Care . The Power Of Information: Giving People Control Of The Health And Care Information They Need. London, UK. 2012. https://www.gov.uk/government/publications/giving‐people‐control‐of‐the‐health‐and‐care‐information‐they‐need

[43] National Information Board . Personalised Health and Care 2020 Using Data and Technology to Transform Outcomes for Patients and Citizens A Framework for Action. London, UK: Department of Health and Social Care; 2014. https://www.gov.uk/government/publications/personalised‐health‐and‐care‐2020

[44] Department of Health and Social Care . The Future Of Healthcare: Our Vision For Digital, Data And Technology In Health And Care. London, UK. 2018. https://www.gov.uk/government/publications/the‐future‐of‐healthcare‐our‐vision‐for‐digital‐data‐and‐technology‐in‐health‐and‐care/the‐future‐of‐healthcare‐our‐vision‐for‐digital‐data‐and‐technology‐in‐health‐and‐care

[45] Rich E , Miah A , Lewis S . Is digital health care more equitable? The framing of health inequalities within England's digital health policy 2010–2017. Sociol Health Illn. 2019;41(S1):31‐49. 31599987 10.1111/1467-9566.12980 PMC7586788

[46] Neter E , Brainin E . eHealth literacy: extending the digital divide to the realm of health information. J Med Internet Res. 2012;14(1):e19. 22357448 10.2196/jmir.1619 PMC3374546

[47] Weiss D , Rydland HT , Oversveen E , Jensen MR , Solhaug S , Krokstad S . Innovative technologies and social inequalities in health: A scoping review of the literature. PLoS One. 2018;13(4):e0195447. 29614114 10.1371/journal.pone.0195447 PMC5882163

[48] Ofcom . Adults: Media Use And Attitudes Report. London, UK: Ofcom; 2019. https://www.ofcom.org.uk/__data/assets/pdf_file/0021/149124/adults‐media‐use‐and‐attitudes‐report.pdf

[49] Lupton D . The digitally engaged patient: Self‐monitoring and self‐care in the digital health era. Soc Theor Health. 2013;11(3):256‐270.

[50] Weiner K , Will C , Henwood F , Williams R . Everyday curation? Attending to data, records and record keeping in the practices of self‐monitoring. Big Data Soc. 2020;7(1):2053951720918275.

[51] Weiner K , Will C . Thinking with care infrastructures: people, devices and the home in home blood pressure monitoring. Sociol Health Illn. 2018;40(2):270‐282. 29464773 10.1111/1467-9566.12590

[52] Langstrup H . Chronic care infrastructures and the home. Sociol Health Illn. 2013;35(7):1008‐1022. 23301691 10.1111/1467-9566.12013

[53] Symons J . ‘We’re not hard‐to‐reach, they are!’ Integrating local priorities in urban research in Northern England: An experimental method. Sociologic Rev. 2017;66(1):207‐223.

[54] Morgan Heather , Thomson Gill , Crossland Nicola , Dykes Fiona , Hoddinott Pat . Combining PPI with qualitative research to engage ‘harder‐to‐reach’ populations: service user groups as co‐applicants on a platform study for a trial. Res Involve Engage. 2016;2(1):7. 10.1186/s40900-016-0023-1 PMC5611582 29062508

[55] Sheikh A , Netuveli G , Kai J , Panesar SS . Comparison of reporting of ethnicity in US and European randomised controlled trials. BMJ. 2004;329(7457):87‐88. 10.1136/bmj.38061.593935.F7 PMC449871 15138158

[56] O’Connor S , Hanlon P , O’Donnell CA , Garcia S , Glanville J , Mair FS . Understanding factors affecting patient and public engagement and recruitment to digital health interventions: a systematic review of qualitative studies. BMC Med Inform Decis Mak. 2016;16(1):120. 27630020 10.1186/s12911-016-0359-3 PMC5024516

