# Supplementary material for: Optimizing a digital intervention for managing blood pressure in stroke patients using a diverse sample: Integrating the person‐based approach and patient and public involvement
Source: Health Expect. 2020 Dec 14;24(2):327–40. doi: 10.1111/hex.13173 (PMC8077154; doi:10.1111/hex.13173)
Supplement: Supplementary file 1 — Appendix S1 [file HEX-24-327-s001.docx]

**Appendix 1: Logic model for BP:Together**

**Problem**

Regular self-monitoring and sharing BP readings with HCP to identify when medication change is needed.

BP:Together booklet

Reporting and prompting tool (SMS, website or app)

BP:Together online tool

Provide face-to-face training and range of digital/non-digital support to self-monitor BP.

Tailored support for people with aphasia.

Manage negative emotion about self-monitoring (Automatic motivation)

↑ Environmental resources (Physical opportunity)

↑ N of medication changes made by HCPs

Appropriate change to antihypertensive medication when average BP readings are above recognised targets.

BP:Together online tool

Emails sent to NHS staff address

Online training tool

↑ Positive beliefs about consequences of changing medication

(Reflective motivation)

Timely, relevant prompts to inform HCP of patient readings and recommended action

Facilitate HCP access to digital intervention

↑ Environmental resources (Physical opportunity)

Plan 3 medication changes per patient in advance

Set goals

(Reflective motivation)

↑ Patient self-monitoring BP and sharing readings digitally with HCP

Provide rationale and evidence from credible sources re BP targets and need for medication change

Discussion with GP at baseline review for reassurance

Poorly controlled BP post-stroke/TIA due to insufficient medication

Reduce systolic BP at 12 months

**Patient**

**HCP**

**Intervention**

**techniques**

**Intervention processes**

**TDF (COM-B)**

**Purported mediators**

**Primary Outcome**

↑ Patient adherence to HCP medication change/

medication self-management

Provide evidence and rationale for BP targets and need for medication change.

Provide information to reassure HCPs that stroke patients are able to self-monitor and self-manage BP

Reassure patients about medication change at baseline review

↑ Positive beliefs about consequences of changing medication

(Reflective motivation)

Provide opportunity for patient to initiate medication change when average BP readings are above target

↑ Beliefs about capability to self-monitor

(Psychological capability)

Provide positive, clear feedback on BP readings

Include a graph to show progress over time

Manage expectations re BP variability

Offer HCP support for reassurance if concerned

Encourage support from significant others where necessary

↑ Social influences

(Social opportunity)

Provide reminder prompts to self-monitor and send BP readings

Allow flexibility in monitoring schedule and option to stop daily reminders to promote acceptability

Practice session to use BP monitor and send readings.

↑ Skills (Physical capability)

**Intervention targets**

**Intervention components**

↑ Environmental resources (Physical opportunity)
